# Thalamic inputs to dorsomedial striatum are involved in inhibitory control: evidence from the five-choice serial reaction time task in rats

**DOI:** 10.1007/s00213-017-4627-4

**Published:** 2017-04-28

**Authors:** Jasjot Saund, Daniel Dautan, Claire Rostron, Gonzalo P. Urcelay, Todor V. Gerdjikov

**Affiliations:** 1 Department of Neuroscience, Psychology and Behaviour, University of Leicester, Leicester, LE1 9HN UK; 2 Center for Molecular and Behavioral Neuroscience, Rutgers University, 197 University Ave, Newark, NJ 07102 UK; 3 Department of Life, Health and Chemical Sciences, Open University, Milton Keynes, MK7 6AA UK

**Keywords:** Thalamostriatal pathway, Dorsomedial striatum, Parafascicular thalamic nucleus, 5-choice serial reaction time task

## Abstract

**Rationale:**

Corticostriatal circuits are widely implicated in the top-down control of attention including inhibitory control and behavioural flexibility. However, recent neurophysiological evidence also suggests a role for thalamic inputs to striatum in behaviours related to salient, reward-paired cues.

**Objectives:**

Here, we used designer receptors exclusively activated by designer drugs (DREADDs) to investigate the role of parafascicular (Pf) thalamic inputs to the dorsomedial striatum (DMS) using the five-choice serial reaction time task (5CSRTT) in rats.

**Methods:**

The 5CSRTT requires sustained attention in order to detect spatially and temporally distributed visual cues and provides measures of inhibitory control related to impulsivity (premature responses) and compulsivity (perseverative responses). Rats underwent bilateral Pf injections of the DREADD vector, AAV2-CaMKIIa-HA-hM4D(Gi)-IRES-mCitrine. The DREADD agonist, clozapine N-oxide (CNO; 1 μl bilateral; 3 μM) or vehicle, was injected into DMS 1 h before behavioural testing. Task parameters were manipulated to increase attention load or reduce stimulus predictability respectively.

**Results:**

We found that inhibition of the Pf-DMS projection significantly increased perseverative responses when stimulus predictability was reduced but had no effect on premature responses or response accuracy, even under increased attentional load. Control experiments showed no effects on locomotor activity in an open field.

**Conclusions:**

These results complement previous lesion work in which the DMS and orbitofrontal cortex were similarly implicated in perseverative responses and suggest a specific role for thalamostriatal inputs in inhibitory control.

## Introduction

Adapting to changing environments requires the allocation of attentional resources including inhibition of inappropriate responding to allow efficient goal-directed behaviour. These functions have traditionally been associated with the corticostriatal network; however, emerging evidence suggests that corticostriatal inputs are subject to thalamic modulation in the striatum (Bradfield et al. 2013a, b).

In rodents, excitotoxic lesions of the dorsomedial striatum (DMS) produce deficits in inhibitory control measured in the five-choice serial reaction time task (5CSRTT), which allows the assessment of both attentional and inhibitory control (Robbins 2002). DMS lesions increase premature and perseverative responding, reflecting deficits in impulsivity and compulsivity, respectively (Rogers et al. 2001). In line with these inhibitory deficits, lesions of the orbitofrontal cortex (OFC) (Chudasama et al. 2003), an area which projects to the DMS in a topographic manner (Schilman et al. 2008), increase perseverative responses in the 5CSRTT. This parallels the effects of OFC and DMS lesions in reversal learning tasks, which also result in an increase in perseverative responses after contingency reversal (Boulougouris et al. 2007; Castañé et al. 2010; Ragozzino et al. 2002).

In addition to receiving inputs from the OFC (Schilman et al. 2008), the DMS is also a target for thalamic inputs (Groenewegen and Berendse 1994), primarily from intralaminar thalamic nuclei (ILN), composed of the centromedian (CM) and parafascicular (Pf) nuclei. Primate studies suggest the CM-Pf complex may provide striatum with behaviourally relevant information (Kimura et al. 2004; Matsumoto et al. 2001; Minamimoto and Kimura 2002); this is supported by imaging studies in humans showing activation of the ILN during an attention-demanding task (Kinomura et al. 1996). Rat lesion studies also implicate Pf in behavioural flexibility. Thus, similar to the deficits observed with OFC and DMS lesions in reversal learning, Pf lesions also impair T-maze reversal learning (Brown et al. 2010) and lead to increased lever pressing after contingency degradation of an instrumental response (Bradfield et al. 2013a). These studies suggest a possible role of the Pf in modulating striatal activity when performance is assessed under changing conditions. However, the specific contribution of this circuit to attentional and inhibitory control has not been isolated experimentally. To address this, here, we characterised the effect of chemogenetic inhibition of the Pf-DMS pathway in the 5CSRTT under task conditions where either stimulus predictability was degraded or attentional load was increased. Stimulus unpredictability has previously been shown to decrease inhibitory control (Chudasama et al. 2003). Inhibition of the Pf-DMS pathway was achieved by use of designer receptors exclusively activated by designer drugs (DREADDs). DREADDs are G protein-coupled receptors that are only activated by their otherwise inert ligand, clozapine N-oxide (CNO), which shows high receptor specificity (Alexander et al. 2009; Armbruster et al. 2007; Wess et al. 2013). Here, the use of a G_i_-coupled DREADD allowed the inhibition of Pf-DMS projections which is thought to be due to synaptic silencing (Alexander et al. 2009; Armbruster et al. 2007). We found that inhibition of this projection significantly increased perseverative responses (a failure of inhibitory control linked to compulsivity) when stimulus predictability was reduced but had no effect on accuracy, even under increased attentional load.

## Methods

### Animals

Ten male Long Evans adult rats, bred within the University of Leicester Central Research Facility, were housed in groups of two or three in a reverse lit room (lights on between 7 pm and 7 am) with constant temperature (22 °C) and humidity (50%). Ad libitum food was restricted to 1–2 h/day on weekdays and was freely available on weekends. Weights were monitored and remained at ∼90% of free-feeding weight. Behavioural testing was carried on weekdays during the dark phase. All experimental and surgical procedures were done in compliance with the Animals (Scientific procedures) Act 1986, UK.

### Apparatus

Training was carried out in four operant chambers [Med Associates, Fairfield, VT; 30 × 31 × 24 cm (height × width × depth); prod. no. ENV-008] placed in sound-attenuated, ventilated cubicles. One side of the chambers comprised the five-choice panel, curved concavely with five apertures with stimulus lights at the back (prod. no. ENV-115A-07V). The other side contained a recessed food magazine, a magazine light and a house light. Entries into all apertures including the food magazine were monitored by a photocell infrared beam. A data acquisition board (National Instruments PCI-6229) interfaced with the operant chambers and custom-written software were used for experimental control and behavioural data acquisition (Labview, National Instruments, Austin, TX, USA). Dustless precision pellets were used as rewards (45 mg, Product No F0021, Bioserv Dustless Precision Pellets, Frenchtown NJ).

### Procedure

#### Behavioural paradigm

The 5CSRTT, as previously described by Robbins (2002), involves the presentation of a visual stimulus in one of five apertures. Rats were required to attend to these apertures and respond with a nose poke into the aperture in which the stimulus was presented. Each session, lasting 40 min or 100 trials (whichever occurred first), began with the delivery of a reward pellet and the illumination of the house light and the magazine light. To initiate the trial, the rat was required to nose-poke into the magazine, after which the magazine light was extinguished, and the inter-trial interval (ITI) began. After the ITI, the light stimulus was illuminated in a random aperture on the five-choice panel. A correct response was recorded if the rat nose-poked into the illuminated aperture within a set time (the limited hold). This triggered the illumination of the magazine light and the release of a sugar pellet from the magazine. A response into the wrong aperture (an incorrect response), a response during the ITI (a premature response) or no response within the limited hold (an omission) were punished with a 5-s timeout in which all lights including the house light were turned off.

Initial training was carried out with longer stimulus durations and limited holds and shorter ITIs which were gradually adjusted based on individual performances to reach target parameters which were stimulus duration (SD), 1.5 s; limited hold (LH), 5 s; ITI, 5 s and timeout 5 s. Virus injection and cannula implantation were carried out once animals achieved 80% accuracy (% correct responses out of total number of responses).

Two weeks following surgery, rats were retrained to criterion and tested on two challenges in the paradigm. The attentional challenge consisted of presenting trials with shorter SDs (1 vs. 2 s, presented randomly within session; initial pre-training had used an SD of 1.5 s) in order to increase attentional load. LH and ITI were fixed at 5 s. A second challenge to test inhibitory control consisted of a varied ITI duration within each session to reduce stimulus predictability (Chudasama et al. 2003). The ITIs were: 4.5, 6, 7.5 and 9 s as used previously (Chudasama et al. 2003). LH was fixed at 5 s and SD at 1.5 s. For each challenge, animals were tested over four consecutive days alternating between vehicle and drug injection days (two of each in total). CNO or vehicle was injected 1 h prior to testing sessions (see ‘Central Infusions’) (Aldrin-Kirk et al. 2016).

#### Performance measures

Accuracy was expressed as the percentage of correct responses (%correct trials/attempted trials). Omissions were calculated as the percentage of omitted trials out of the total number of trials. Premature and perseverative responses were used as measures of inhibitory control. Premature responses were counted as responses made during the ITI and calculated as a percentage out of all trials. Perseverative responses were defined as nose-pokes into one of the five apertures after a correct response was made but before collection of the reward as has been reported previously (Chudasama et al. 2003) and expressed as a percentage out of the number of correct trials. Response latencies were calculated as the time taken to respond to a stimulus on correct trials. Latency to collect reward was taken as the time between reward delivery and collection of the reward.

### Surgical procedures

#### DREADD injection

Rats were anaesthetised with 4% *v*/*v* isoflurane (Schering-Plough) in O_2_ and maintained between 2 and 3%. The animal was mounted in a stereotaxic frame, and the head was adjusted so that lambda and bregma were aligned on the same horizontal plane. To prevent corneal desiccation, Lacri-Lube Eye Ointment (Allergan, Westport, Ireland) was applied to the eyes. A homoeothermic heat pad (Harvard Apparatus, Boston, Massachusetts, USA) was used to maintain body temperature between 36 and 37 °C. Glucose (5%, 3 ml/h, s.c.) was given via an infusion pump (Intec, K.D, Scientific, Holliston, Massachusetts, USA) for the duration of the surgery. Small craniotomies were made directly above the virus injection sites; Pf target coordinates were −4.2 mm AP, ±1.8 mm ML and −5.5 mm DV (Paxinos and Watson 2005). Dorsal ventral coordinates were taken from the dura; all other coordinates were from bregma.

**Fig. 1:**
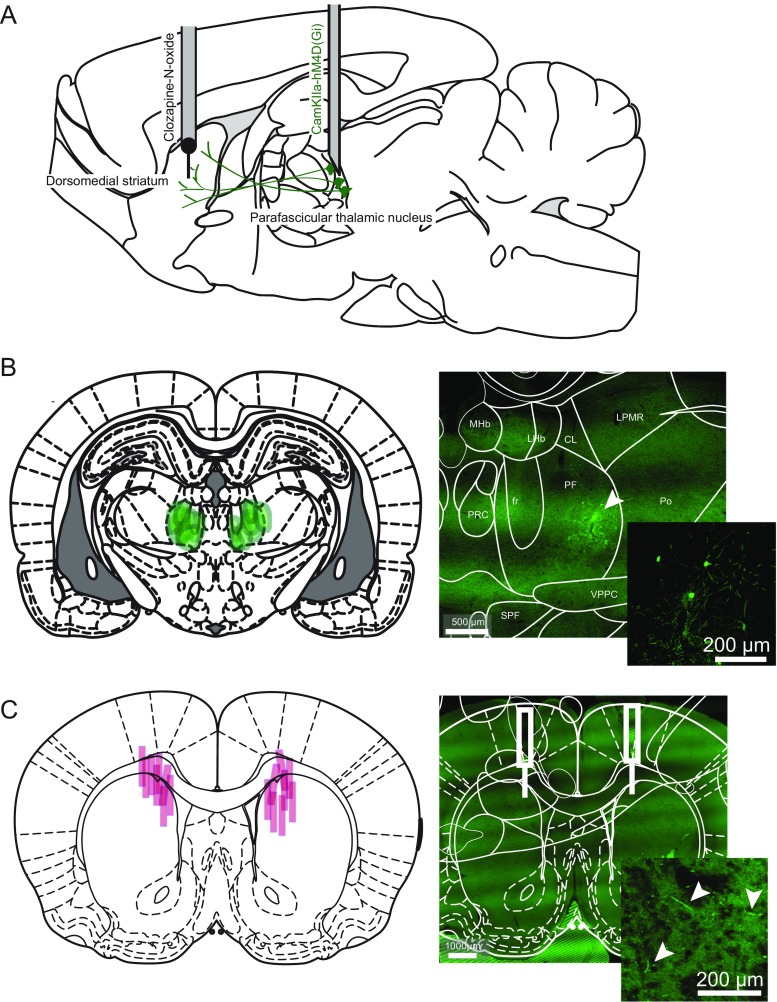
Surgical preparation and immunohistochemical verification of anatomical targets. **a** Sagittal view of the rat brain indicating sites of virus injection (AAV2-CaMKIIa-HA-hM4D(Gi)-IRES-mCitrine) in the Pf and CNO in the DMS. **b** Left panel shows diagram of coronal view of the intralaminar thalamic nuclei with *green eclipses* indicating individual virus diffusion areas. Right panel shows confocal image of a thalamic slice indicating midline and intralaminar thalamic nuclei with GFP indicating expression of the DREADD (*arrow*). Inset shows Pf in higher magnification. **c** Left panel shows a coronal view of striatum indicating individual cannula tracks. Right panel shows a confocal image of striatum with injection tracks indicated. Inset shows DMS in higher magnification to show presence of GFP indicating DREADD expression in Pf-DMS axonal processes (marked with *arrows*)

A 1-μl Hamilton syringe (Neuros 7000 Series) was mounted on an infusion pump (Pump 11 Elite Nanomite, Harvard Apparatus, Holliston, MA,USA) and filled with 0.8 μl of virus, AAV2-CaMKIIa-HA-hM4D(Gi)-IRES-mCitrine (University of North Carolina – Vector Core). The virus was injected at a rate of 50 nl/min into Pf, and the injection needle was left in place for a further 10 min to allow diffusion. This injection was done bilaterally with the syringe cleaned and reloaded with virus between injections (Fig. 1a).

#### Cannula implantation

Cannula guides were implanted in DMS to allow the central injections of the DREADD agonist, CNO. These were done in the same surgery as the virus injections. Coordinates used for DMS were −0.5 mm AP, ±1.8 mm ML and −3.0 mm DV (Paxinos and Watson 2005). Dorsal ventral coordinates were taken from the dura; all other coordinates were from bregma.

Custom-made 23-gauge cannulas were inserted bilaterally into the striatal coordinates and fixed in place using light-curing dental cement (Flowable Composite, Henry Schein; Gillingham, UK). Stainless steel stylets were placed in the cannulas to prevent blockage.

#### Post-operative care

A non-steroidal anti-inflammatory (Carprieve, 5 mg/kg; S.C.; Norbrook Laboratories Ltd.; Corby, UK) was administered 1–2 h before recovery and twice a day for 5 days post-surgery. An antibiotic (Baytril, 2.5%, 0.2 ml/kg; S.C.; Bayer; Leverkusen, Germany) was given immediately after recovery and twice daily for 5 days after surgery.

### Central infusions

One hour prior to testing, 1 μl of 3 μM CNO or vehicle (artificial CSF + DMSO) was injected through the DMS cannula guides using a 10-μl Hamilton precision syringe connected to polyethylene tubing with a 30-gauge steel injector on the end. The injector protruded beyond the cannula length by 1 mm. CNO was injected at a rate of 0.5 μl/min, and the injector was left in place for another minute to allow for drug diffusion. The CNO dose used here was similar to published work (Stachniak et al. 2014). The drug was injected 1 h prior to testing following work from the Roth lab showing that behavioural effects of CNO administration increase gradually, are robust after an hour and persist for many hours after administration (Alexander et al. 2009).

### Locomotion activity

Locomotor activity was assessed in an open-top black Plexiglas box (52 wide × 52 long × 40 high) using a digital camera and the ANY-MAZE tracking software (Stoelting, IL, USA). Each session consisted of 40 min, and all animals completed two sessions (CNO vs vehicle, counterbalanced) on two separate days. One hour prior to testing, the animals were injected with 1 μl of either CNO or artificial CSF.

### Histology

DREADD expression in Pf cell bodies and axon terminals in the DMS was verified using labelling with green fluorescent protein. Rats were terminally anaesthetised with pentobarbital (250 mg/kg; IP) and perfused with 5% formaldehyde. Brains were extracted and kept in saline for 2 days at 4 °C and subsequently transferred to 0.05% sodium azide (*w*/*v* in saline) and kept at 4 °C until sectioning. Coronal sections of the striatum and the thalamus were cut at 50-μm thickness using a vibratome (VT1200S; Leica). Sections were incubated in a blocking solution consisting of 10% normal donkey serum (NDS) in PBS containing 1% Triton X-100 for a minimum of 1 h. Sections were then washed three times in PBS and incubated with antibody against green florescent protein (GFP, 1:1000, raised in rabbit, Invitrogen, A21311) in 1% normal donkey serum (NDS), 0.03% Triton X-100 in PBS. Sections were mounted in slices and protected with Vectashield. Z-Stack fluorescent images were obtained with a confocal microscope (LSM-510, Zeiss) using the 504-nm filter for Alexa Fluor-488 at two different magnifications, ×4 (0.4 numerical aperture) and ×20 (1.2 numerical aperture), at 2048 × 2048 resolution and a digital camera (Hamamatsu ORCA-ER digital Camera, Hamamatsu Photonics K.K.). The brightness and contrast of the images were subsequently adjusted in ImageJ. Virus diffusion area and injector tracks were reported on a single projection slice.

### Data analysis

Time stamps of all behavioural events in the 5CSRTT were recorded using custom-made LabVIEW software, and behavioural data were analysed using within-subject analysis of variance (ANOVA) with drug (CNO vs. vehicle) and parameter value (ITI or SD duration) as the within-subject variables. Statistical analyses were performed using SPSS (IBM SPSS, Somers, NY, USA). In several cases, drug treatment was critically found to have no effect on omissions or in locomotor activity. In these cases, we computed Bayes factors for use in supporting the null hypothesis using a freely available Bayes factor calculator (http://pcl.missouri.edu/bayesfactor). In all cases, the scale of *r* was set up to 1.0 which serves as a natural benchmark (Rouder et al. 2009).

## Results

### Histology

Verification of DREADD expression was carried using green fluorescent protein. Virus expression was detected in both Pf cell bodies and DMS axon terminals. Verification of DMS cannula implantation sites was also confirmed histologically (Fig. 1b, c). Two animals with an off-target virus injection and a DMS cannula site respectively were removed from subsequent analyses. Thus, the analyses reported below are based on *N* = 8 rats undergoing repeated-measures testing.

### Effect of CNO on 5CSRTT performance measures

Prior to testing, animals reached an average accuracy of 86.5 ± 1.5% (mean ± SEM) and an average of 20.0 ± 4.2% omissions. CNO did not have an effect on the total number of trials that the rats completed on the varied ITI or the shorter SD challenges [*F*(1, 7) = 0.08, *p* = 0.788 and *F*(1, 7) = 0.25, *p* = 0.617, respectively].

#### Variable ITI challenge

To determine the effect of CNO on response measures under decreased stimulus predictability, we injected CNO or vehicle on 2 days (2 CNO and 2 artificial CSF days, counterbalanced) where ITI duration was varied within-session: 4.5, 6, 7.5 or 9 s. This experiment was analysed using a 4 × 2 × 2 (ITI duration x drug x repetition) within-subjects ANOVA. The variable repetition was added since two CNO and two vehicle days were recorded. Due to a violation of the sphericity assumption, the Greenhouse-Geisser correction was applied. Reduced stimulus predictability affected response measures as expected: we saw an increase in omissions [*F*(1.488, 10.413) = 9.97, *p* = 0.006] and an increase in premature responding [*F*(3, 18) = 91.47, *p* < 0.001] (see Table 1). These patterns replicate previous work (Chudasama et al. 2003).

**Table 1:** Performance measure averages (mean ± SEM) for the four ITI durations

Performance measures	ITI(s)			
	4.5	6	7.5	9
% Accuracy	90.3 (±1.9)	89.2 (±1.9)	87.0 (±2.9)	85.3 (±2.9)
% Omissions*	14.0 (±1.9)	16.1 (±1.7)	16.7 (±1.9)	27.2 (±3.9)
% Premature*	3.9 (±1.6)	18.8 (±3.8)	32.8 (±4.6)	53.0 (±5.1)
% Perseverative	10.0 (±3.8)	12.3 (±3.7)	14.2 (±4.3)	11.2 (±4.4)
Response latency(s)	0.90 (±.05)	0.89 (±.07)	0.86 (±.06)	0.86 (±.06)
Latency to collect reward(s)	2.03 (±.20)	2.10 (±.23)	2.13 (±.21)	2.01 (±.20)

*Significant main effect of the respective measure (*p* < 0.05)

**Fig. 2:**
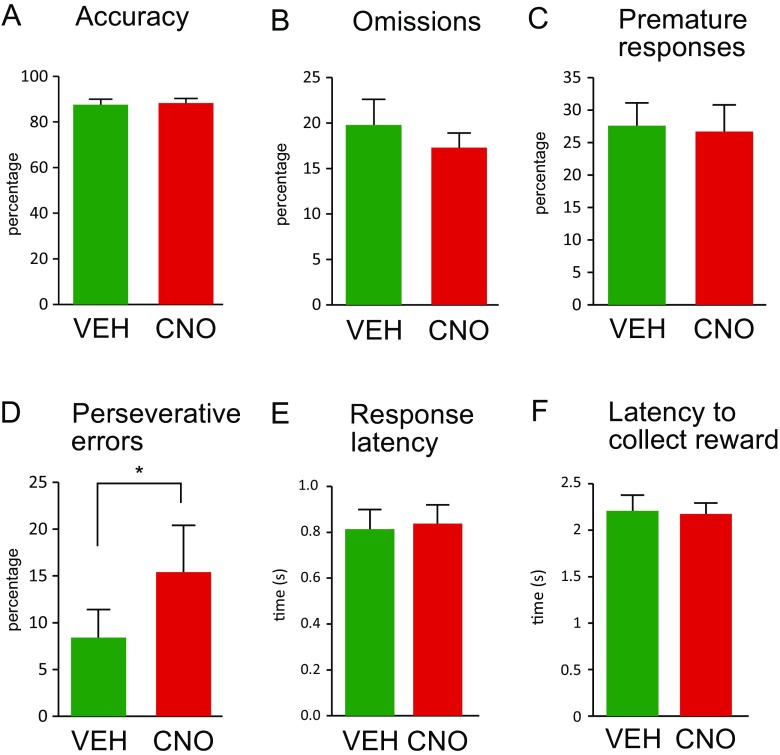
Inhibiting Pf-DMS projections with CNO injections in dorsomedial striatum produce a specific perseverance deficit when stimulus predictability is degraded by varying ITI duration. No significant effects of CNO are seen in response accuracy (**a**), percentage omissions (**b**) or premature responses (**c**). In contrast, CNO injection increases perseverative errors (**d**). Response latency (**e**) or latency to collect reward (**f**) is not affected. *Significant difference from vehicle, *p* < .05 (within-subject ANOVA). *Error bars* indicate SEM. Data is averaged across ITIs and the two repetitions

CNO had no significant effect on accuracy [*F*(1, 7) = 0.32, *p* = 0.590], omissions [*F(*1, 7) = 0.98, *p* = 0.356] or premature responses [*F*(1, 7) = 0.98, *p* = 0.356] (Fig. 2a–c). A Bayesian analysis conducted on omission trials found that these data are 2.53 times more likely to be observed under the null hypothesis predicting no difference between CNO and vehicle. However, CNO did increase significantly the percentage of perseverative responses [*F*(1, 7) = 7.13, *p* = 0.032] (Fig. 2d) without producing a drug × ITI interaction [*F*(3, 21) = 0.72, *p* = 0.550]. CNO treatment did not affect response latency or latency to collect rewards [*F*(1, 7) = 0.12, *p* = 0.743 and *F*(1, 7) = 1.44, *p* = 0.269, respectively] (Fig. 2e, f). There were no CNO × ITI interactions on any of the performance measures. Further, there were no effects of repetition or interactions involving repetition.

#### Shorter SD challenge

A second experiment assessed the effect of CNO on performance measures under increased attentional load using two stimulus durations within-session (1 vs 2 s; initial training had been carried out using a stimulus duration of 1.5 s). CNO or vehicle was injected on 2 days (2 CNO and 2 vehicle days, counterbalanced), and the experiment was analysed using a 2 × 2 × 2 (stimulus duration × drug × repetition) within-subjects ANOVA. The variable repetition was added since two CNO and two vehicle days were recorded.

As expected, accuracy was lower for 1 vs. 2 s stimuli [mean ± SEM 87.8 ± 2.9% vs 89.6 ± 2.4%; *F*(1, 7) = 12.83, *p* = 0.009], and percentage omissions were higher [20.4 ± 2.7% vs 14.2% ± 1.9%, *F*(1, 7) = 28.94, *p* = 0.001]. Also, as expected response latencies were shorter for 1-s stimuli than for 2-s stimuli [0.88 ± 0.06 s vs 0.98 ± 0.06 s; *F(*1, 7) = 6.87, *p* = 0.034]. Stimulus duration did not affect perseverative responses [*F*(1, 7) = 0.90, *p* = 0.373].

CNO did not appear to affect response parameters in this challenge of the task: CNO had no significant effect on accuracy [*F*(1, 7) = 0.02, *p* = 0.869], percentage of omitted trials [*F*(1, 7) = 0.02, *p* = 0.883], premature responses [*F*(1, 7) = 0.885, *p* = 0.378], perseverative responses [*F*(1, 7) = 0.05, *p* = 0.827], response latency [*F*(1, 7) = 0.28, *p* = 0.612] or latency to collect rewards [*F*(1, 7) = 0.28, *p* = 0.613]. There were no CNO × SD interactions on either of the performance measures. A Bayesian analysis conducted on omission trials found that these data are 3.87 times more likely to be observed under the null hypothesis predicting no difference between CNO and vehicle.

Finally, our analysis of perseverative responses followed previous work on response inhibition (Chudasama et al. 2003; Rogers et al. 2001) and included nose-pokes into any nose hole (i.e. indiscriminately). Additional analyses when considering only nose-pokes in the rewarded nose hole produced no drug effects or interactions involving drug treatment (*p*s > .25 for all effects, drug × ITI × repetition within-subjects ANOVA for the variable ITI challenge). The same was true when considering the shorter SD challenge (*p*s > .30), except we note that the *p* value for the effect of drug in the latter analysis was *p* = .08 [*F*(1, 7) = 4.20].

### Locomotion activity

**Fig. 3:**
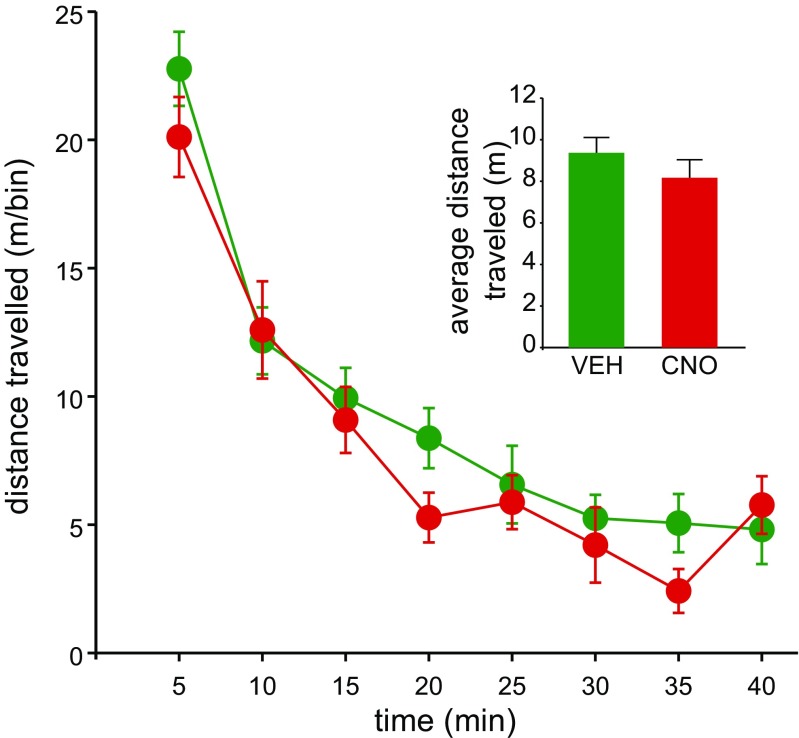
Local administration of CNO into dorsomedial striatum produces no deficits in locomotor activity. Locomotion was measured as distance travelled (mean ± SEM) in an open field after vehicle (artificial CSF) or CNO administration recorded in 5-min bins. The inset shows distance travelled (mean ± SEM) averaged for the whole 40-min session

To test for non-specific locomotor effects of CNO injections in DMS, we recorded activity in an open field after CNO vs. vehicle injections administered on 1 of 2 days (counterbalanced). Distance travelled (m) was binned into eight 5-min time windows. A 2 × 8 (drug × bin) within-subject ANOVA revealed no significant effect of drug or drug × bin interaction [*F*(1, 7) = 1.17, *p* = 0.314 and *F*(7, 49) = 1.17, *p* = 0.339, respectively]. A Bayesian analysis found that these data are 2.34 times more likely to be observed under the null hypothesis predicting no difference between CNO and vehicle. As expected, locomotor activity showed significant habituation over time [*F*(7, 49) = 49.41, *p* < 0.001] (Fig. 3).

## Discussion

The present study investigated the role of the Pf-DMS pathway in inhibitor control and attention. We used chemogenetic inhibition to temporarily and specifically inactivate the Pf-DMS pathway and assessed performance on behavioural measures in the 5CSRTT. Viral expression in Pf cell bodies and processes in DMS was confirmed immunohistochemically. Task parameters were systematically manipulated to increase attentional load or reduce stimulus predictability, respectively. The main finding of the study is that inhibition of the Pf-DMS projection significantly increases perseverative responses (a failure of inhibitory control related to compulsivity) compared to vehicle when stimulus predictability was reduced but had no effect on accuracy, even under increased attentional load.

It is unlikely that non-specific effects of CNO injections into DMS could explain the observed pattern of results. Firstly, gross locomotor effects are unlikely because we did not observe an effect on the total number of trials completed, response latencies, reward retrieval latencies and ambulatory activity in an open field. This conclusion was supported by Bayesian analyses which suggested that the null hypothesis (i.e. no effect) was more strongly supported by the data. While our sample size was similar to previous reports of within-subject pharmacological manipulations in the five-choice serial reaction time task (Murphy et al. 2012; Pezze et al. 2007; Robinson et al. 2008), the null findings on these additional measures may require further investigation given that a priori effect sizes are not available.

Further, with respect to the application of the DREADD methodology, while one recent study reports retroconversion of CNO to clozapine in rats and related behavioural effects (MacLaren et al. 2016), we do not believe this is likely to provide an alternative explanation for our specific findings: multiple publications demonstrate that clozapine does not affect perseverative responses in the 5-CSRTT with systemic doses as high as 2.5 and 5 mg/kg (Baviera et al. 2008; Carli et al. 2011; Turner et al. 2013). The absence of an effect on perseverative responses is dissociable from an increase in omissions reported in the latter two papers with doses of 2.5 and 5 mg/kg, respectively. The opposite pattern was observed in our work: we saw an increase in perseverative responses but no effect on omissions. Thus, it is unlikely that clozapine mediates the behavioural effect seen here. Overall, non-specific effects of CNO cannot explain the pattern of results obtained in the current study.

Analysis of specific behavioural parameters as a function of task manipulations confirmed the robustness of the protocol. As found previously, varying ITI duration increased both premature responses and omissions (Chudasama et al. 2003). Similarly, in the shorter SD challenge, the decrease in accuracy and higher number of omissions as well as decreased response latency with shorter stimuli was not surprising and has previously been reported (Amitai and Markou 2011; Bari et al. 2008; Guillem et al. 2011).

In the current study, we found a specific role for the Pf-DMS projection in perseverative responding. Perseverance on the 5CSRTT is an indication of compulsivity (Dalley et al. 2011): compulsive behaviour is described as a repetitive inappropriate behaviour that persists, often despite negative outcomes (Dalley et al. 2011). Perseverant behaviours can arise in a variety of contexts including those requiring behavioural flexibility and response inhibition. Here, perseverative entries into the five apertures after a correct response are not only unrewarded but are also disadvantageous in that they delay the collection of the reward. This effect was specific to variable ITI sessions but did not correlate with ITI length, suggesting that it is driven by the temporal unpredictability of the response cues. In contrast, DMS excitotoxic lesions produce other deficits in addition to perseverative responses, including premature responses and reduced accuracy, suggesting a more general deficit related to both inhibitory and attentional control. DMS receives strong prefrontal innervation from OFC (Schilman et al. 2008), and OFC-lesioned animals show increased premature responses, omissions and perseverative responses. With extensive training, both the omission and premature deficits are reduced in OFC-lesioned rats, but the perseverative deficits persist, suggesting a deficit parallel to the one obtained using pharmacogenetic inhibition of the Pf-DMS projection in the current study (Chudasama et al. 2003). These observations further suggest that DMS integrates top-down OFC and bottom-up Pf inputs, and disruption of either of these produces perseverative behaviour.

The thalamostriatal behavioural deficit observed here shows high specificity since other performance indices were not affected by inhibiting the pathway in the varied ITI challenge, nor were any of performance measures affected in the shorter SD challenge. This strengthens the suggestion that the Pf-DMS pathway is specifically involved in behavioural flexibility as opposed to attentional control. This is also consistent with Pf lesion work which implicates this structure in behavioural flexibility. For example, Pf-lesioned rats were unimpaired when initially learning an action-outcome association in a lever-pressing instrumental task and were sensitive to outcome devaluation. However, when the contingencies changed (i.e. were degraded), they were unable to update the action-outcome association and thus failed to reduce responding to the degraded lever (Bradfield et al. 2013a). Furthermore, Pf inactivation also causes regressive errors in T-maze reversal learning task (Brown et al. 2010). These regressive errors reflect an inability to maintain the new action-outcome contingency, further supporting the role of the Pf in behavioural flexibility.

Additional support for the role of Pf in behavioural flexibility arises from evidence implicating this structure in modulation of striatal cholinergic interneurons (CINs) (Brown et al. 2010). Striatal CINs appear to mediate, to a large extent, Pf inputs to the DMS. Not only are they shown to respond to salient reward predicting stimuli (Doig et al. 2014; Matsumoto et al. 2001), but this response is also shown to transiently suppress the cortically driven medium spiny neurons (MSNs). Furthermore, both DMS and Pf receive common cholinergic projections from the cholinergic brainstem (Dautan et al. 2014) that has also been showed to modulate dopamine release in the striatum (Dautan et al. 2016a, b). Whether and to what extent cholinergic and other intervening interneuron and/or neuromodulator systems may mediate the behavioural effects reported here should be investigated in future studies [see also Faust et al. (2016)].

Finally, we have to acknowledge that the current study did not specify the type of Pf cells targeted with CNO. Studies suggest that Pf striatal projections are mainly glutamatergic (Smith et al. 2014) and that glutamic acid decarboxylase (GAD, the synthetic enzyme for GABA) immunoreactivity in rat Pf is weak or absent (Bentivoglio et al. 1991). However, the current data clearly does not contribute to this discussion and these previous anatomical results may have to be complemented with modern viral-based approaches. Further, our observation of behavioural effects 1 h after intracerebral CNO administration are in line with previous work (Mahler et al. 2014; Stachniak et al. 2014). However, we have to acknowledge the possibility that the time interval between CNO administration and behavioural testing may potentially affect the functioning of the transfected neurons independently or secondarily to the inhibition presumed here based on previous work.

In summary, the present study examined the role of thalamic input to the dorsomedial striatum in attention and behavioural flexibility using the 5CSRTT. We manipulated task parameters to either increase attentional load or reduce stimulus predictability to challenge behavioural flexibility; we exploited the selectivity afforded by the DREADD approach to specifically interrogate the contribution of the Pf-DMS pathway to these processes and found that thalamic projections are implicated in perseverative responses, indicating a role of this projection in inhibitory control. Our results suggest that the behavioural role of dorsomedial striatum must be considered in the context of thalamostriatal projections in addition to previously described prefrontal inputs.

## References

[CR1] Aldrin-Kirk P, Heuer A, Wang G, Mattsson B, Lundblad M, Parmar M (2016). DREADD modulation of transplanted DA neurons reveals a novel parkinsonian dyskinesia mechanism mediated by the serotonin 5-HT6 receptor. Neuron.

[CR2] Alexander GM, Rogan SC, Abbas AI, Armbruster BN, Pei Y, Allen JA (2009). Remote control of neuronal activity in transgenic mice expressing evolved G protein-coupled receptors. Neuron.

[CR3] Amitai N, Markou A (2011). Comparative effects of different test day challenges on performance in the 5-choice serial reaction time task. Behav Neurosci.

[CR4] Armbruster BN, Li X, Pausch MH, Herlitze S, Roth BL (2007). Evolving the lock to fit the key to create a family of G protein-coupled receptors potently activated by an inert ligand. Proc Natl Acad Sci U S A.

[CR5] Bari A, Dalley JW, Robbins TW (2008). The application of the 5-choice serial reaction time task for the assessment of visual attentional processes and impulse control in rats. Nat Protoc.

[CR6] Baviera M, Invernizzi RW, Carli M (2008). Haloperidol and clozapine have dissociable effects in a model of attentional performance deficits induced by blockade of NMDA receptors in the mPFC. Psychopharmacology.

[CR7] Bentivoglio M, Spreafico R, Minciacchi D, Macchi G (1991). GABAergic interneurons and neuropil of the intralaminar thalamus: an immunohistochemical study in the rat and the cat, with notes in the monkey. Exp Brain Res.

[CR8] Boulougouris V, Dalley JW, Robbins TW (2007). Effects of orbitofrontal, infralimbic and prelimbic cortical lesions on serial spatial reversal learning in the rat. Behav Brain Res.

[CR9] Bradfield LA, Bertran-Gonzalez J, Chieng B, Balleine BW (2013). The thalamostriatal pathway and cholinergic control of goal-directed action: interlacing new with existing learning in the striatum. Neuron.

[CR10] Bradfield LA, Hart G, Balleine BW (2013b). The role of the anterior, mediodorsal, and parafascicular thalamus in instrumental conditioning. Front Syst Neurosci 7:51 10.3389/fnsys.2013.00051 PMC3793176 24130522

[CR11] Brown HD, Baker PM, Ragozzino ME (2010). The parafascicular thalamic nucleus concomitantly influences behavioral flexibility and dorsomedial striatal acetylcholine output in rats. J Neurosci.

[CR12] Carli M, Calcagno E, Mainini E, Arnt J, Invernizzi RW (2011). Sertindole restores attentional performance and suppresses glutamate release induced by the NMDA receptor antagonist CPP. Psychopharmacology.

[CR13] Castañé A, Theobald DE, Robbins TW (2010). Selective lesions of the dorsomedial striatum impair serial spatial reversal learning in rats. Behav Brain Res.

[CR14] Chudasama Y, Passetti F, Rhodes S, Lopian D, Desai A, Robbins T (2003). Dissociable aspects of performance on the 5-choice serial reaction time task following lesions of the dorsal anterior cingulate, infralimbic and orbitofrontal cortex in the rat: differential effects on selectivity, impulsivity and compulsivity. Behav Brain Res.

[CR15] Dalley JW, Everitt BJ, Robbins TW (2011). Impulsivity, compulsivity, and top-down cognitive control. Neuron.

[CR16] Dautan D, Huerta-Ocampo I, Witten IB, Deisseroth K, Bolam JP, Gerdjikov T (2014). A major external source of cholinergic innervation of the striatum and nucleus accumbens originates in the brainstem. J Neurosci.

[CR17] Dautan D, Bay HH, Bolam JP, Gerdjikov TV, Mena-Segovia J (2016a) Extrinsic sources of cholinergic innervation of the striatal complex: a whole-brain mapping analysis. Front Neuroanat 10:1 10.3389/fnana.2016.00001 PMC4722731 26834571

[CR18] Dautan D, Souza AS, Huerta-Ocampo I, Valencia M, Assous M, Witten IB et al (2016b) Segregated cholinergic transmission modulates dopamine neurons integrated in distinct functional circuits. Nat Neurosci 19:1025-33 10.1038/nn.4335 PMC5086413 27348215

[CR19] Doig NM, Magill PJ, Apicella P, Bolam JP, Sharott A (2014). Cortical and thalamic excitation mediate the multiphasic responses of striatal cholinergic interneurons to motivationally salient stimuli. The Journal of neuroscience : the official journal of the Society for Neuroscience.

[CR20] Faust TW, Assous M, Tepper JM, Koos T (2016). Neostriatal GABAergic interneurons mediate cholinergic inhibition of spiny projection neurons. J Neurosci.

[CR21] Groenewegen J, Berendse W (1994). The specificity of the ‘nonspecific’ midline and intralaminar thalamic nuclei. Trends Neurosci.

[CR22] Guillem K, Bloem B, Poorthuis RB, Loos M, Smit AB, Maskos U (2011). Nicotinic acetylcholine receptor beta2 subunits in the medial prefrontal cortex control attention. Science.

[CR23] Kimura M, Minamimoto T, Matsumoto N, Hori Y (2004). Monitoring and switching of cortico-basal ganglia loop functions by the thalamo-striatal system. Neurosci Res.

[CR24] Kinomura S, Larsson J, Gulyás B, Roland PE (1996). Activation by attention of the human reticular formation and thalamic Intralaminar nuclei. Science.

[CR25] MacLaren DA, Browne RW, Shaw JK, Radhakrishnan SK, Khare P, España RA *et al.* (2016) Clozapine n-oxide administration produces behavioral effects in long–evans rats: implications for designing DREADD experiments. *eneuro* 3: ENEURO. 0219–16.2016 10.1523/ENEURO.0219-16.2016 PMC5089539 27822508

[CR26] Mahler SV, Vazey EM, Beckley JT, Keistler CR, McGlinchey EM, Kaufling J (2014). Designer receptors show role for ventral pallidum input to ventral tegmental area in cocaine seeking. Nat Neurosci.

[CR27] Matsumoto N, Minamimoto T, Graybiel AM, Kimura M (2001). Neurons in the thalamic CM-pf complex supply striatal neurons with information about behaviorally significant sensory events. J Neurophysiol.

[CR28] Minamimoto T, Kimura M (2002). Participation of the thalamic CM-Pf complex in attentional orienting. J Neurophysiol.

[CR29] Murphy ER, Fernando AB, Urcelay GP, Robinson ES, Mar AC, Theobald DE (2012). Impulsive behaviour induced by both NMDA receptor antagonism and GABAA receptor activation in rat ventromedial prefrontal cortex. Psychopharmacology.

[CR30] Paxinos G, Watson C (2005). The rat brain in stereotaxic coordinates.

[CR31] Pezze M, Dalley JW, Robbins TW (2007). Differential roles of dopamine D1 and D2 receptors in the nucleus accumbens in attentional performance on the five-choice serial reaction time task. Neuropsychopharmacology.

[CR32] Ragozzino ME, Jih J, Tzavos A (2002). Involvement of the dorsomedial striatum in behavioral flexibility: role of muscarinic cholinergic receptors. Brain Res.

[CR33] Robbins T (2002). The 5-choice serial reaction time task: behavioural pharmacology and functional neurochemistry. Psychopharmacology.

[CR34] Robinson ES, Dalley JW, Theobald DE, Glennon JC, Pezze MA, Murphy ER (2008). Opposing roles for 5-HT2A and 5-HT2C receptors in the nucleus accumbens on inhibitory response control in the 5-choice serial reaction time task. Neuropsychopharmacology.

[CR35] Rogers RD, Baunez C, Everitt BJ, Robbins TW (2001). Lesions of the medial and lateral striatum in the rat produce differential deficits in attentional performance. Behav Neurosci.

[CR36] Rouder JN, Speckman PL, Sun D, Morey RD, Iverson G (2009). Bayesian t tests for accepting and rejecting the null hypothesis. Psychon Bull Rev.

[CR37] Schilman EA, Uylings HB, Galis-de Graaf Y, Joel D, Groenewegen HJ (2008). The orbital cortex in rats topographically projects to central parts of the caudate–putamen complex. Neurosci Lett.

[CR38] Smith Y, Galvan A, Ellender TJ, Doig N, Villalba RM, Huerta-Ocampo I (2014). The thalamostriatal system in normal and diseased states. Front Syst Neurosci.

[CR39] Stachniak TJ, Ghosh A, Sternson SM (2014). Chemogenetic synaptic silencing of neural circuits localizes a hypothalamus→midbrain pathway for feeding behavior. Neuron.

[CR40] Turner KM, Young JW, McGrath JJ, Eyles DW, Burne TH (2013). Cognitive performance and response inhibition in developmentally vitamin D (DVD)-deficient rats. Behav Brain Res.

[CR41] Wess J, Nakajima K, Jain S (2013). Novel designer receptors to probe GPCR signaling and physiology. Trends Pharmacol Sci.

